# Medical rescue of naval combat: challenges and future

**DOI:** 10.1186/s40779-015-0048-z

**Published:** 2015-08-26

**Authors:** Hai Jin, Li-Jun Hou, Xiao-Bing Fu

**Affiliations:** Department of Neurosurgery, Changzheng Hospital, Second Military Medical University, Shanghai, 200433 China; Department of Key Laboratory of Wound Repair and Regeneration of PLA, College of Life Sciences, General Hospital of PLA, Beijing, 100853 China

**Keywords:** Naval combat, Medical rescue challenges, Rescue highlights, Future development

## Abstract

There has been no large-scale naval combat in the last 30 years. With the rapid development of battleships, weapons manufacturing and electronic technology, naval combat will present some new characteristics. Additionally, naval combat is facing unprecedented challenges. In this paper, we discuss the topic of medical rescue at sea: what challenges we face and what we could do. The contents discussed in this paper contain battlefield self-aid buddy care, clinical skills, organized health services, medical training and future medical research programs. We also discuss the characteristics of modern naval combat, medical rescue challenges, medical treatment highlights and future developments of medical rescue at sea.

## Background

There is no doubt that Spratly Islands and Diaoyu Islands belong to China. Some countries have disputed over the territory for centuries, and recent increased tension has made the area a substantial issue in Asia. Based on this issue, the possibility of military conflict or naval combat is a problem for the involved countries. In naval combat history, the latest large-scale naval battle was the 1982 Falklands Battle between UK and Argentina, which caused a total of 907 deaths and 1843 injuries [1]. Based on Chinese records of naval combat in 1974 and 1988 between China and Vietnam in the South China Sea [2–4], the weapons used in these limited-scale combats included short-range artilleries, guns and grenades. Despite the rapid development of battleships, weapons manufacturing and electronic technology in the last 30 years, naval combat will present many new characteristics.

Established in 1871, U.S. Navy Medicine Health Care now consists of five distinct “Corps”: Medical Service Corps, Nurse Corps, Medical Corps, Dental Corps and Hospital Corps. Each corps consists of personnel specializing in a particular health care field [5]. Additionally, the U.S. has established navy medicine departments around the global to support the Navy and Marine Corps. In Asia, U.S. navy medicine centers exist in Japan, Cambodia, Singapore and Vietnam. These factors indicate the importance of medical rescue at sea [6].

## Challenges of medical rescue at sea

### Environmental

The battlefield tends to be in the ocean, far from land or shoreline. Casualty rescue would be extremely difficult during the sustained fighting environment in the battlefield. Some characteristics of the seawater include cold temperature, hypertonicity and pathogenic bacteria, which cause additional damage to the casualty [7–9]. For example, compared with simple firearm wounds, the healing time of firearm wounds after seawater immersion can be delayed [10].

### Weapons

Widespread use of high-speed and precision-guided underwater explosive weapons or missiles can cause more complex and multiple forms of injuries [11]. Blast damage is the leading cause of death on the modern battlefield, especially in navy combat [12]. For tactical purposes, non-explosive weapons such as infrasound weapons, laser weapons, electromagnetic weapons and microwave weapons can be used on the battlefield.

### Combatant distribution

For the purpose of mobility, concealment and ammunition load, surface ships and marines’ cabins are usually designed to be narrow and small [13]. Because of the densely distributed combatants in the cabins, there could be substantial casualties once attacked. Additionally, the casualties in various cabins would present various types of trauma.

## Difficulties of medical rescue at sea

### Casualty distribution

Combatants in surface battleships and submarines are concentrated and dense. Once attacked, there would be substantial casualties at the same time, and the doctors would have difficulty evaluating all casualties in a short period of time. In some situations, once combatants are dropped in the seawater, an unpredictable distribution of combatants would occur because of the waves and the battlefield’s blasts. In this type of dispersed distribution, it is very difficult to search or salvage the casualty, thereby delaying treatment. The Health Department of People’s Liberation Army (PLA) invented an electronic casualty location system that can help search for casualties on land, but it has not been well tested in the naval setting [14, 15].

### Complex injuries

The blast of explosive weapons can cause injuries to multiple sites of the body at the same time, such as head trauma, extremity fractures, spine injury and chest or abdomen trauma. The initial injury mechanism is acceleration damage from the blast, and the consequent injury mechanism is deceleration damage from hitting the bulkhead or instruments inside the cabins [16]. Some weapons will cause combined injuries such as blast injury, burn injury, seawater immersion injury and decompression injury, etc. [17]. These combined injuries cause extremely complex clinical manifestations and are difficult to treat.

### Diagnostic and transport difficulties

Well-equipped hospital ships, such as PLA NO.866 hospital ship and USNS Mercy, usually maintain a particular distance from the battlefield [18]. Sometimes, doctors on the battleships diagnose the casualty at the place where the damage occurred by simple medical equipment or even only by clinical symptoms [19, 20]. Casualty transport from surface battleships to hospital ships mainly depends on suspended transporters on rescue ships or limited helicopters [21]. Submarines rarely use surface transport due to their undercover and combat mission requirements. Therefore, we must recognize that casualty transport from battleships to hospital ships or land-based hospitals are very difficult during war [22].

## Medical rescue highlights

### Self-Aid Buddy Care (SABC)

SABC encompasses basic life support and limb-saving techniques to help casualty or injured personnel survive in medical emergencies until medical help is available. Generally, SABC requires that injury judgments must be accurate, measures must be quick and everyone must do their best to make the casualty stable [23]. The airway, breathing, circulation, disability and exposure (ABCDE) approach still plays a classic and practical role in the immediate assessment and treatment of the critical casualty [24].

### Damage control

In the pre-hospital treatment of a critical casualty, the best method is damage control surgery to avoid further deterioration [25]. Doctors correct hypothermia, acidosis, coagulopathy and other fatal failures at the same time of initial resuscitation [26]. In encountering multiple associated injuries, priority attention should be given to high mortality situations such as shock, bleeding and brain trauma [27, 28]. In U.S. military medicine, the Forward Surgical Team (FST) has been tested to be a very efficient group of doctors who can complete damage control surgery independently [29–31].

### Efficient transport

For those urgent or critical casualties who need further medical treatment, it is important to arrange air transport as soon as possible. Forward doctors report the patient’s information to the higher-level hospital before transportation so doctors can prepare efficiently to ensure the continuous treatment after transportation [32]. Various vehicles (military ships, military planes or civilian ships) should be used to rescue as many lives as possible during war.

## Medical rescue: future tasks

### SABC training

The training of all combatants is critical for meeting our current requirements and preparing for challenges. The topics of SABC training encompass administrative overview, anatomy and physiology, communicable diseases/universal precautions, airway management, recognition and control of bleeding, shock management, dressings, bandaging, fractures, splinting, heat/cold related injuries, burn injuries, victim assessment and patient transportation/litter movement [33, 34].

### Trauma protection and new weapons research

Protection is important for all types of trauma [35, 36]. To relieve blast shock in naval combat, it is necessary to set anti-shock devices such as anti-shock chairs and seat belts in the possible positions in the cabins of battleships. Cabins should have anti-collision materials installed on bulkheads and equipment surfaces to avoid secondary collision injuries. Combatants should be equipped with impact resistance helmets and cushioning shoes. As the mechanism of new weapons injuries remains unclear, it is imperative for military medicine researchers to continue investigating the weapons and their effects [37].

### Combination of military and civilian medical forces

It would be efficient to establish a medicine command center that can organize military and civilian medical forces during war. Closer to battle, Forward Surgical Teams (FSTs) could play a substantial role [30]. New diagnostic equipment could be invented, such as portable intracranial hematoma diagnostic equipment and portable electronic monitor devices. To be closer to casualties, there should be enough Dock Hospital or Combat Support Hospitals (CSH) on nearby coasts. Additionally, civilian medical forces also undertake important tasks in casualty care, blood supply, drug supply, etc.

### Injury mechanism of seawater

Characteristics of seawater injuries include drowning, cold temperature, hypertonicity and pathogenic bacteria. A study found 21 forms of bacteria from 85 strains in the Nansha district [38]. The average bacterial number was 336.60 ± 160.79 cfu/ml. These bacteria were all highly susceptible to 16 antibiotics. Another study found 34 types of bacteria in the Southeast littoral [39]. The susceptibilities of the bacteria to 21 antibiotics were tested. These investigations are important for the prevention and therapy of bacterial infections related to seawater. It is essential to create a practical rewarming plan for hypothermia in casualties salvaged from seawater [40, 41].

## Conclusion

Medical rescue is a complicated and challenging topic. The topic involves many indispensable aspects that should be considered during peace times. We must clearly understand the challenges we face and the weaknesses in our current medical system. Future work should include training and research. The future development of trauma prevention and portable equipment used in medical rescue at sea must be powerful enough to save more people.

## Abbreviations

SABC: Self-aid buddy care
ABCDE: Airway, breathing, circulation, disability and exposure
FST: Forward surgical team
CSH: Combat support hospital
